# The Role of Microbes in the Nutrition of Detritivorous Invertebrates: A Stoichiometric Analysis

**DOI:** 10.3389/fmicb.2016.02113

**Published:** 2017-01-04

**Authors:** Thomas R. Anderson, David W. Pond, Daniel J. Mayor

**Affiliations:** ^1^National Oceanography CentreSouthampton, UK; ^2^Scottish Association for Marine Science, Natural Environment Research CouncilOban, UK

**Keywords:** detritus, microbial loop, stoichiometry, trophic upgrading, polyunsaturated fatty acids, mesopelagic zone

## Abstract

Detritus represents an important pool in the global carbon cycle, providing a food source for detritivorous invertebrates that are conspicuous components of almost all ecosystems. Our knowledge of how these organisms meet their nutritional demands on a diet that is typically comprised of refractory, carbon-rich compounds nevertheless remains incomplete. “Trophic upgrading” of detritus by the attached microbial community (enhancement of zooplankton diet by the inclusion of heterotrophic protozoans) represents a potential source of nutrition for detritivores as both bacteria and their flagellated protistan predators are capable of biosynthesizing essential micronutrients such as polyunsaturated fatty acids (PUFAs). There is however a trade-off because although microbes enhance the substrate in terms of its micronutrient content, the quantity of organic carbon is diminished though metabolic losses as energy passes through the microbial food web. Here, we develop a simple stoichiometric model to examine this trade-off in the nutrition of detritivorous copepods inhabiting the mesopelagic zone of the ocean, focusing on their requirements for carbon and an essential PUFA, docosahexaenoic acid (DHA). Results indicate that feeding on microbes may be a highly favorable strategy for these invertebrates, although the potential for carbon to become limiting when consuming a microbial diet exists because of the inefficiencies of trophic transfer within the microbial food web. Our study highlights the need for improved knowledge at the detritus-microbe-metazoan interface, including interactions between the physiology and ecology of the associated organisms.

## Introduction

The production of dead and decaying particulate organic matter (“detritus” hereafter) may account for as much as 56% of primary production when averaged across a range of ecosystems (Cebrián and Duarte, 1995). This flux of detritus thereby constitutes a significant term in the global carbon cycle (Ciais et al., 2013) and is a major conduit through which organic matter is transported both within and between ecosystems (Bartels et al., 2012). It also provides sustenance to countless detritivorous invertebrates, which we loosely interpret as any animal that has a trophic association with dead organic matter, including organismal egesta. Detritus-detritivore interactions influence the potential for carbon sequestration in both terrestrial and aquatic environments. Understanding the interface between living and dead organic matter is therefore a prerequisite to improving predictions of global biogeochemical cycles and climate (Burd et al., 2016; Luo et al., 2016).

Detritus is mainly composed of refractory compounds such as structural polysaccharides (Mann, 1988; Kiem and Kögel-Knabner, 2003), but is depleted in micronutrients such as amino acids and fatty acids (Cowie and Hedges, 1996; Pokarzhevskii et al., 1997; Mayor et al., 2011) that are considered essential for the growth of metazoan animals (Müller-Navarra et al., 2000; Anderson et al., 2004; Sampedro et al., 2006; Larsen et al., 2016). The nutritional challenge facing detritivores may, however, be mitigated by the presence of microorganisms that colonize the detrital substrate (Moran and Hodson, 1989; Turley and Mackie, 1994). Detritivores actively ingest this detritus-associated microbial community which, unlike the basal substrate, is readily absorbed and provides a rich source of micronutrients (Bärlocher and Kendrick, 1975; Phillips, 1984; Lawrence et al., 1993; Koski et al., 2005). Indeed, a key functional characteristic of many detritivorous invertebrates is their propensity to shred or fragment detritus (Anderson and Sedell, 1979; Iversen and Poulsen, 2007), an activity that has been proposed to stimulate the production of microbial biomass by increasing the surface area of the substrate, so-called “microbial gardening” (Fenchel, 1970; Mayor et al., 2014). The resulting uplift in the nutritional content of detritus represents a form of “trophic upgrading,” a term which originates from the marine literature and refers to the enhancement of zooplankton growth by the inclusion of micronutrient-rich heterotrophic protozoans in an otherwise herbivorous diet (Klein Breteler et al., 1999). Relying on microbes as a primary source of nutrition does, however come at an energetic cost because their gross growth efficiencies are typically <30 % (Del Giorgio and Cole, 1998) and the majority of organic carbon in the detrital substrate is therefore lost during the trophic upgrading process. Detritivorous invertebrates thus face a trade-off between consuming a high quality, low quantity diet that is rich in microbes versus the low quality, high quantity detritus (Mayor et al., 2014).

Here, we use a simple stoichiometric model to examine the extent to which invertebrates maximize growth by incorporating microbes into their diet, using detritivorous zooplankton in the mesopelagic zone (MPZ) of the ocean as a case study. The MPZ extends from the base of the sunlit (euphotic) zone down to ~1000 m and many of the resident organisms are primarily sustained by an estimated global detrital flux of 5–12 Gt C yr^−1^ (Henson et al., 2011). The depth at which organic matter is remineralized within the MPZ influences the residence time of carbon in the oceans and hence global climate (Kwon et al., 2009). Sinking detrital particles in the MPZ exhibit the characteristic poor nutritional status described above, having undergone stripping of the most desirable compounds by bacteria and/or multiple ingestion events by zooplankton (Podgórska and Mundryk, 2003; Wilson et al., 2008). The resulting substrate is thus largely devoid of essential micronutrients such as amino or fatty acids (Wakeham et al., 1997; Fileman et al., 1998; Schneider et al., 2003). We suggest that the problem of obtaining sufficient nutrition may be felt acutely by detritivorous zooplankton that permanently reside in the MPZ, e.g., copepods of the genus *Oithona* that are ubiquitous throughout the world ocean (Gallienne and Robins, 2001; Dahms et al., 2015). Members of this genus are well known to interact with detrital particles (Gonzalez and Smetacek, 1994; Iversen and Poulsen, 2007), particularly in the mesopelagic (Suzuki et al., 2003). Organisms inhabiting the MPZ experience high hydrostatic pressure and low temperatures, both of which negatively affect the functioning of cellular membranes (Hazel and Williams, 1990). Zooplankton overcome these difficulties by increasing the relative abundance of the essential polyunsaturated fatty acid, docosahexaenoic acid (DHA), in their membranes (Pond et al., 2014). Copepods and other highly motile zooplankton also possess myelin-like sheathes around their nerve axons to facilitate rapid escape responses (Raymont et al., 1974; Davis et al., 1999) and DHA has been suggested to be an important component of the associated sphingomyelin lipid pool (Scott et al., 2002). The model presented herein has C and DHA as currencies and is used to examine the trade-off for detritivorous zooplankton when consuming a high quantity, low DHA:C diet (detritus) versus a nutritionally-upgraded diet of microbial biomass present in low quantity, but with a high DHA:C ratio. Our analysis, which is underpinned by empirical data from a number of sources, highlights the need for improved understanding of food web processes in the mesopelagic, including the associated physiology of the resident organisms.

## Model description

### Equations

**Figure 1:**
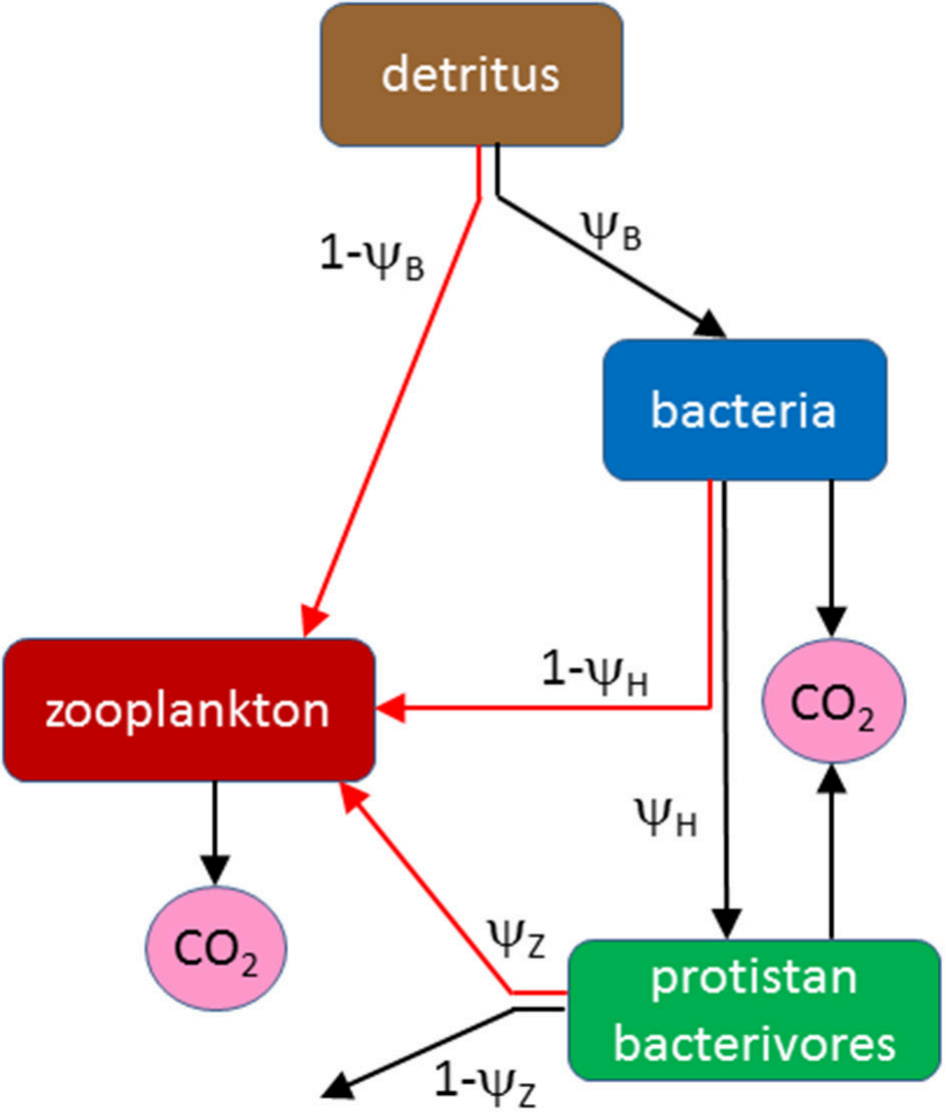
**Flow diagram of the model showing pathways of organic matter between detritus, bacteria, protistan bacterivores and zooplankton, as specified by parameters ψ_**B**_, ψ_**H**_, and ψ_**Z**_**. Black arrows represent C-only flows, red arrows involve both C and DHA (involving stoichiometric calculations).

The model is a steady-state flow analysis of the detrital food web in the MPZ of the ocean, including colonization of detritus by microbes (particle-attached bacteria and protistan bacterivores) and their consumption by detritivorous zooplankton (Figure 1; lists of model variables and parameters are provided in Tables 1, 2). The main focus is the growth of zooplankton and its stoichiometric regulation by C and DHA. The baseline currency of the model is C from which flows are calculated throughout the food web as a whole. Zooplankton growth, on the other hand, is calculated from stoichiometric equations involving both C and DHA. Fixed ratios (model parameters) are specified for DHA:C in detritus, bacteria and bacterivores which, in conjunction with predicted C cycling throughout the food web, permits an assessment of the roles of C and DHA in limiting the growth of zooplankton (depending on the relative availability of each food type to their diet). It is thus possible to examine the potential trade-off between consuming a high quantity, low quality diet (detritus with a low DHA:C ratio) versus a low quantity, high quality diet (microbes with a high DHA:C ratio). In this context, it is useful to define the two end-members of the nutritional spectrum: a “detritivorous pathway” and a “microbial pathway.” The former represents consumption of the non-living detrital substrate, whereas the microbial pathway consists of a diet solely of microbes. Our default assumption is that detritivorous zooplankton selectively ingest protistan bacterivores on the basis of their motility. The microbial pathway therefore represents a diet consisting solely of these organisms and excludes particle-attached bacteria. The sensitivity of predicted zooplankton growth to whether or not bacteria constitute a food source will nevertheless be investigated by including the possibility of ingesting bacteria in the model structure and parameterization.

**Table 1:** **Model variables**.

**Variable**	**Definition**	**Unit of measure**
F_D_	Entry flux of D into system	mol C m^−3^ d^−1^
A_C, det_	Absorption C: detrit. path	mol C m^−3^ d^−1^
A_DHA, det_	Absorption DHA: detrit. path	mmol DHA m^−3^ d^−1^
A_C, mic_	Absorption C: microb. path	mol C m^−3^ d^−1^
A_DHA, mic_	Absorption DHA: microb path	mmol DHA m^−3^ d^−1^
G_B_	Bacterial production	mol C m^−3^ d^−1^
G_H_	Bacterivore production	mol C m^−3^ d^−1^
G_Z_	Zooplankton production	mol C m^−3^ d^−1^

**Table 2:** **Model parameters**.

**Parameter**	**Definition**	**Default value**	**Unit of measure**
θ_D_	DHA:C, detritus	0.21	mmol DHA mol C^−1^
θ_Z_	DHA:C, zooplankton	1.76	mmol DHA mol C^−1^
θ_B_	DHA:C, bacteria	0.08	mmol DHA mol C^−1^
θ_H_	DHA:C, bacterivores	1.40	mmol DHA mol C^−1^
ω_B_	bacteria GGE	0.12	dimensionless
β_H_	AE, bacterivores on bacteria	0.72	dimensionless
k_H_	max. NPE, bacterivores: C	0.44	dimensionless
β_ZC_	AE, zooplankton on D: C	0.1	dimensionless
β_ZDHA_	AE, zooplankton on D: DHA	0.1	dimensionless
β_ZBH_	AE, zooplankton on B,H	0.72	dimensionless
k_ZC_	max. NPE, zooplankton: C	0.36	dimensionless
k_ZDHA_	max. NPE, zoopl.: DHA	0.9	dimensionless
ψ_B_	partitioning D to bacteria	0.1	dimensionless
ψ_H_	partitioning B to bacterivores	1.0	dimensionless
ψ_Z_	partitioning H to zoopl.	0.8	dimensionless

The stoichiometric calculations of zooplankton growth assume that these animals are unable to synthesize DHA *de novo* (Bell et al., 2007) in which case this essential fatty acid can be treated in the same way as elements such as C, N and P when using theoretical stoichiometry to analyze limitation of growth (Anderson and Pond, 2000). Bacteria and bacterivores are, on the other hand capable of synthesizing essential acids, including DHA, *de novo* (Klein Breteler et al., 1999; Russell and Nichols, 1999; Fang et al., 2002) and so their growth is calculated assuming that limitation is by C.

Detritus provides the foundation of the mesopelagic food web, specified as an input flux to the model, *F*_*D*_ (mol C m^−3^ d^−1^). The detrital substrate is acted on by either particle-attached bacteria (fraction ψ_*B*_) or by zooplankton (fraction 1-ψ_*B*_). The latter gives rise to the detritivorous pathway, which we consider first. Ingested C and DHA following this pathway, i.e., from direct consumption of non-living detritus by zooplankton, are subject to absorption efficiencies (AEs) β_*ZC*_ and β_*ZDHA*_ in which case quantities of absorbed C and DHA, *A*_*C, det*_ and *A*_*DHA, det*_, are:

(1) $$\begin{array}{r} A_{C,\det}=\left(1-\psi_{B}\right)\beta_{ZC}F_{D} \end{array}$$

(2) $$\begin{array}{r} A_{DHA,\det}=\left(1-\psi_{B}\right)\beta_{ZDHA}\theta_{D}F_{D} \end{array}$$

where θ_*D*_ is the DHA:C ratio in detritus (excluding microbes within the detrital matrix).

The alternative is for detritivores to obtain nutrition by consuming microbes, the “microbial pathway,” which necessitates predicting the availability of bacteria and protistan bacterivores deriving from trophic transfer within the food web. Bacteria utilize detritus with growth efficiency ω_*B*_, from which their growth, *G*_*B*_, is:

(3) $$\begin{array}{r} G_{B}=\psi_{B}\omega_{B}F_{D} \end{array}$$

The fate of bacteria in the model is either consumption by protistan bacterivores within the particle-attached food web (fraction ψ_*H*_) or zooplankton (fraction 1-ψ_*H*_); note that our default assumption is that of zero consumption by zooplankton, i.e., ψ_*H*_ = 1. The growth of the bacterivores, *G*_*H*_, is calculated as the product of ingestion (ψ_*H*_*G*_*B*_), absorption efficiency (for C; parameter β_*H*_) and net production efficiency (NPE; the fraction of absorbed C allocated to growth; parameter *k*_*H*_):

(4) $$\begin{array}{r} G_{H}=\psi_{H}\beta_{H}k_{H}G_{B} \end{array}$$

Total ingestion of C by zooplankton via the microbial pathway is the sum of that on bacteria, (1-ψ_*H*_)*G*_*B*_, and protistan bacterivores, ψ_*Z*_*G*_*H*_ (fraction ψ_*Z*_ of bacterivore production is utilized by zooplankton), with corresponding intake of DHA calculated from the DHA:C ratios of these food sources (θ_*B*_ and θ_*H*_ for bacteria and protistan bacterivores, respectively). The resulting quantities of absorbed C and DHA following the microbial pathway, *A*_*C, mic*_ and *A*_*DHA, mic*_, are then:

(5) $$\begin{array}{r} A_{C,mic}=\beta_{ZBH}\left(\left(1-\psi_{H}\right)G_{B}+\psi_{Z}G_{H}\right) \end{array}$$

(6) $$\begin{array}{r} A_{DHA,mic}=\beta_{ZBH}\left(\left(1-\psi_{H}\right)\theta_{B}G_{B}+\psi_{Z}\theta_{H}G_{H}\right) \end{array}$$

where β_*ZBH*_ is absorption efficiency for zooplankton on bacterivores (applied equally to C and DHA).

Zooplankton growth can now be calculated using established stoichiometric equations (e.g., Anderson and Hessen, 1995) that compare the relative availability of C and DHA in absorbed substrates, as supplied by both the detritivorous and microbial pathways. If C is limiting then growth, *G*_*Z*_ (mol C m^−3^ d^−1^), is:

(7) $$\begin{array}{r} G_{Z}\left(C\right)=k_{ZC}\left(A_{C,\det}\;+\;A_{C,mic}\right) \end{array}$$

where parameter *k*_*ZC*_ is the maximum NPE for C (maximum *k*_*ZC*_ occurs when C is limiting; realized *k*_*ZC*_ is lower when DHA is limiting growth because C is then in stoichiometric excess). The corresponding equation for *G*_*Z*_ when DHA is limiting is:

(8) $$\begin{array}{r} G_{Z}\left(DHA\right)=k_{ZDHA}\left(A_{DHA,\det}+A_{DHA,mic}\right)/\theta_{Z} \end{array}$$

where *k*_*ZDHA*_ is maximum net production efficiency for DHA and θ_*Z*_ is the DHA:C ratio in zooplankton biomass. Realized growth is then then the minimum of the calculated C- and DHA-limited rates:

(9) $$\begin{array}{r} G_{Z}=MIN\left[G_{Z}\left(C\right),G_{Z}\left(DHA\right)\right] \end{array}$$

A threshold elemental ratio (TER) can be calculated, $\theta_{A}^{*}$, which is the optimum ratio of DHA and C in absorbed substrates for growth:

(10) $$\begin{array}{r} \theta_{A}^{*}=\frac{k_{ZC}\theta_{Z}}{k_{ZDHA}} \end{array}$$

With parameters as in Table 2 (*k*_*ZC*_ = 0.36, *k*_*ZDHA*_ = 0.9 and θ_*Z*_ = 1.76), calculated $\theta_{A}^{*}$ is 0.70 meaning that optimal growth requires that each mol of absorbed C is accompanied by 0.70 mmol of absorbed DHA.

### Parameterization

Model parameters fall into three categories: those specifying trophic transfer (growth efficiencies), those that define the fractionation of C between the different flow pathways in the model, and the four parameters that define DHA:C ratios in biomass. Starting with the first category, the absorption efficiency of C for zooplankton grazing on detritus, parameter β_*ZC*_, was assigned a low value of 0.1 because of the refractory nature of the substrate (Bärlocher and Kendrick, 1975). The same absorption efficiency was applied to DHA, i.e., β_*ZDHA*_ = 0.1, thereby assuming that zooplankton are unable to selectively extract DHA from the detritus matrix; this parameter will be subject to sensitivity analysis. Living microbes are considerably more amenable to digestion by zooplankton and so the efficiencies with which ingested bacteria and protistan bacterivores are absorbed, parameter β_*ZBH*_ (applied equally to both groups), was assigned a value of 0.72 (Anderson and Tang, 2010). The net production efficiency with which absorbed C is used for growth is well below 1.0 because of the energetic costs of metabolism. We set *k*_*ZC*_ = 0.36 based on a mean gross growth efficiency (GGE) of 0.26 for copepods (Straile, 1997) from which NPE is calculated by dividing through by AE of 0.72 (GGE is the product of AE and NPE). The role of essential fatty acids such as DHA in metabolism is not well known. The simplest assumption is that they are not heavily involved in which case DHA may be utilized for growth with high NPE e.g., *k*_*ZDHA*_ = 0.9 (Anderson and Pond, 2000; Mayor et al., 2009).

Moving on to the microbial food web, a typical BGE for particle-attached bacteria is 0.24 (Anderson and Tang, 2010) but this does not take into account that as much as 50% of the substrate may be lost in dissolved form through solubilization by exoenzymes (Anderson and Tang, 2010; Mayor et al., 2014). The model here does not explicitly represent solubilization losses and therefore, in practical terms, the value of 0.24 should be halved, giving ω_*B*_ = 0.12. The magnitude of BGE is not well understood in marine systems and so this parameter, which sets the inflow of carbon to the microbial pathway, will be the subject of sensitivity testing. Protistan bacterivores graze on the particle-attached bacteria. As for the zooplankton, an absorption efficiency of 0.72 was applied, along with a NPE for C of 0.44 (derived from a GGE of 0.32 for flagellates: Straile, 1997), parameters β_*H*_ and *k*_*H*_, respectively.

Parameters for the fractionation of C via the flow pathways in the food web, ψ_*B*_, ψ_*H*_, and ψ_*Z*_, are not easy to estimate. The first of these, namely the partitioning of detritus usage between particle-attached bacteria (parameter ψ_*B*_, leading to the microbial pathway) and detritivorous zooplankton (1-ψ_*B*_; leading to the detritivorous pathway) was guesstimated at 0.75 by Anderson and Tang (2010) based on the data of Steinberg et al. (2008). An improved estimate of ψ_*B*_ = 0.5 was justified by Mayor et al. (2014), based on data from the North Atlantic. Most of our analysis of the model will focus on the two separate ends of the spectrum of this parameter, i.e., ψ_*B*_ = 0,1, in order to provide a theoretical comparison of the nutritional benefits of the detritivorous and microbial pathways in isolation to each other. Values of ψ_*B*_ that lead to optimal zooplankton nutrition are then calculated, which can be compared to the estimates above. The trophic linkages of the microbial food web on particles are not well known but it is reasonable to expect a tight coupling between bacteria and protistan bacterivores because of their close proximity (Grossart and Ploug, 2001), and thereby a high value of ψ_*H*_. Moreover, it may be that the detritivorous zooplankton selectively ingest protistan bacteriovores on the basis of their motility (Kiørboe, 2011), leaving the bacteria untouched, in which case ψ_*H*_ = 1 (the default value used in our analysis). The fate of flagellate biomass is even less certain. We tentatively assume that, without other obvious predators, the majority of the flagellate loss term is available to support the growth of zooplankton and set ψ_*Z*_ = 0.8.

### Data sources

Studies that concurrently present data on the C and DHA content of marine seston and/or organisms are scarce, and almost non-existent for the MPZ. Parameter values for the DHA:C values in seston biomass, θ_*D*_ = 0.21 mmol mol^−1^ (detritus), θ_*B*_ = 0.08 (bacteria), θ_*H*_ = 1.4 (protistan bacterivores) and θ_*Z*_ = 1.76 (zooplankton) were therefore obtained from a variety of representative sources.

The DHA:C content of detritus (θ_*D*_ = 0.21 mmol mol^−1^) is for seston collected on a pre-combusted GF/F filter (0.7 μm) at a depth of 215 m in the Bellingshausen Sea, Antarctica (Fileman et al., 1998). This likely represents an upper-estimate of this parameter because the sample came from the upper MPZ and the collection method made no attempt to distinguish between non-living detritus and (DHA-rich) organismal biomass. The DHA:C content of particle-attached bacteria (θ_*B*_ = 0.08 mmol mol^−1^) represents an average value derived from various culture studies on deep-sea microbes (θ_*B*_ = 0.11, 0.11, 0.03; Fang et al., 2002, 2003, 2004, respectively). The DHA:C content of protistan bacterivores (θ_*H*_ = 1.4 mmol mol^−1^) is an average value for the heterotrophic dinoflagellate, *Oxyrrhis marina*, reared on the algae *Rhodomonas* sp. (θ_*H*_ = 1.54) and *Dunaliella* sp. (θ_*H*_ = 1.32) (Klein Breteler et al., 1999). An average value for the DHA:C content of zooplankton (θ_*Z*_ = 1.76 mmol mol^−1^) was used based on published data for female copepods of the species *Oithona similis*, collected from between 400 m depth and the surface in Antarctic waters (Pond and Ward, 2011). Interested readers are guided to the relevant citations for further details of individual sample collection and analysis.

## Results

The main focus of the analysis presented herein is a theoretical examination of the two ends of the nutritional spectrum, namely the detritivorous pathway (ψ_*B*_ = 0; zooplankton diet of non-living detritus) and the microbial pathway (ψ_*B*_ = 1; diet consisting solely of protistan bacterivores). This provides the most effective means of examining the trade-off between consuming a high quantity, low quality diet (detritus with a low DHA:C ratio) versus a low quantity, high quality diet (microbes with a high DHA:C ratio). The growth of zooplankton on a mixed diet incorporating both detritus and microbes will be investigated thereafter.

**Figure 2:**
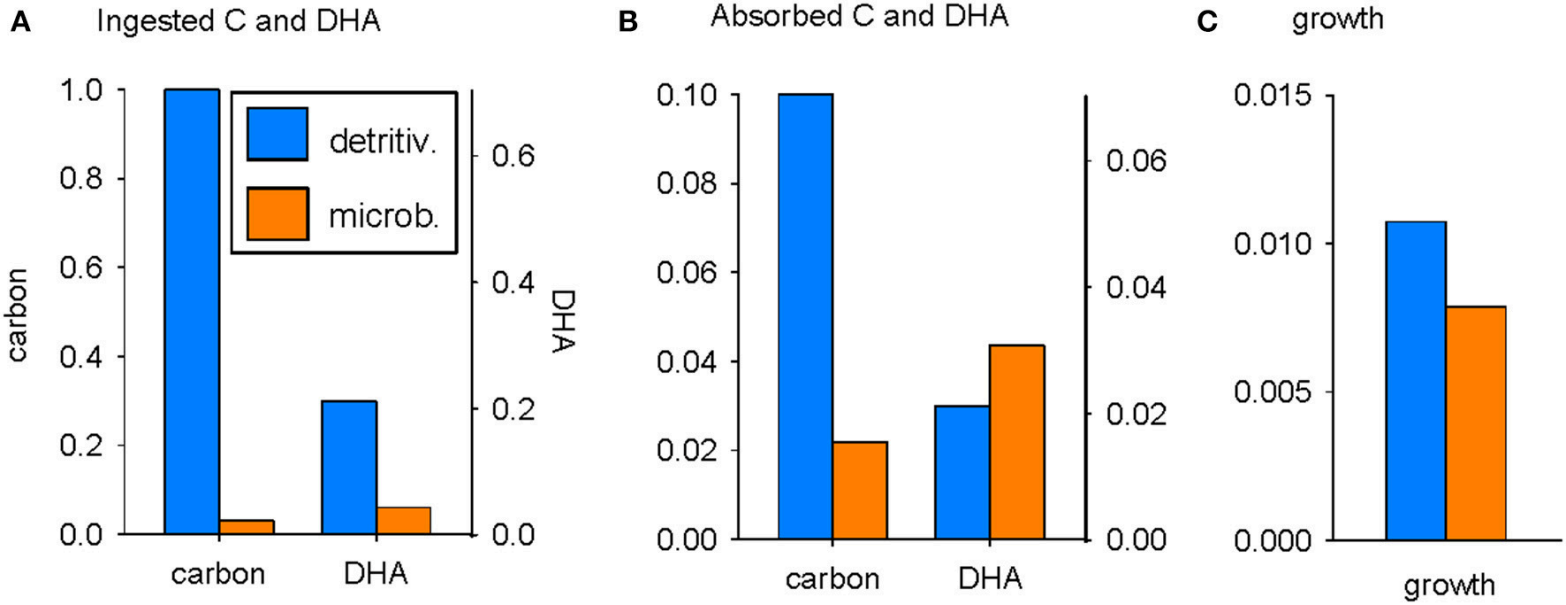
**Utilization of C and DHA by zooplankton following the detritivorous (ψ_**B**_ = 0; blue) and microbial (ψ_**B**_ = 1; orange) pathways: (A)** ingestion, **(B)** absorption, **(C)** growth. *F*_*D*_ = 1 mol C m^−3^ d^−1^; units of ingestion and absorption of C, and growth, are mol C m^−3^ d^−1^; units for ingestion and absorption of DHA are mmol m^−3^ d^−1^. DHA is scaled to the optimum absorption ratio (Equation 10: see text).

The utilization of C and DHA by zooplankton for growth, via ingestion and absorption, is compared for the detritivorous and microbial pathways in Figure 2 (parameters as in Table 2). The detritus flux into the system, *F*_*D*_, was nominally set at 1 mol C m^−3^ d^−1^, facilitating ease of analysis (everything is normalized to an input of 1; there is no need to use an observed value of *F*_*D*_ in order to compare the relative merits of the detritivorous and microbial pathways as a source of nutrition for zooplankton). The supply of C via the detritivorous pathway is plentiful whereas ingestion of C via the microbial pathway is reduced by 97% because of C losses in trophic transfer associated with the growth efficiencies of bacteria and bacterivores (Figure 2A). Perhaps surprisingly, detritus is also predicted to be the most plentiful source of DHA, with intake of 0.21 mmol m^−3^ d^−1^ compared to 0.043 mmol m^−3^ d^−1^ via the microbial pathway (Figure 2A). This is again a consequence of the much diminished stocks of bacterivore biomass compared to detritus and occurs despite the DHA:C ratio being more than six times higher in bacterivores (1.4 in bacterivores versus 0.21 mmol mol^−1^ in detritus). Microbial biomass is, however, absorbed with much higher efficiency than detritus (β_*ZBH*_ = 0.72 versus β_*ZC*_ = β_*ZDHA*_ = 0.1) and so the difference in substrate supply between the two pathways is diminished post-absorption (Figure 2B). The absorbed quantity of DHA is greatest following the microbial pathway (0.031 vs. 0.021 mmol m^−3^ d^−1^) whereas the amount of absorbed C remains considerably lower than in the detritivorous pathway (0.022 vs. 0.1 mol C m^−3^ d^−1^).

The growth of zooplankton depends not only on quantities of absorbed substrates, but also on the net production efficiencies for DHA and C, *k*_*ZDHA*_ and *k*_*ZC*_ respectively, as well as the DHA:C ratio in biomass, θ_*Z*_ (Equations 7, 8). Note that the DHA axes in Figure 2 are scaled to the optimal DHA:C ratio in absorbed substrates ($\theta_{A}^{*}$ = 0.70; Equation 10) so that the potential for growth limitation by C or DHA can be determined by visual comparison of the bar heights for a given trophic pathway. It can be seen that predicted zooplankton growth following the detritivorous pathway is limited by DHA (the blue bar for DHA is lower than that for C in Figure 2B) whereas growth following the microbial pathway is limited by C (the orange bar for C is lower than that for DHA). Overall, the assembled parameter set indicates that growth is greatest following the detritivorous pathway, although the margin is small (0.011 vs. 0.008 mol C m^−3^ d^−1^; Figure 2C).

**Figure 3:**
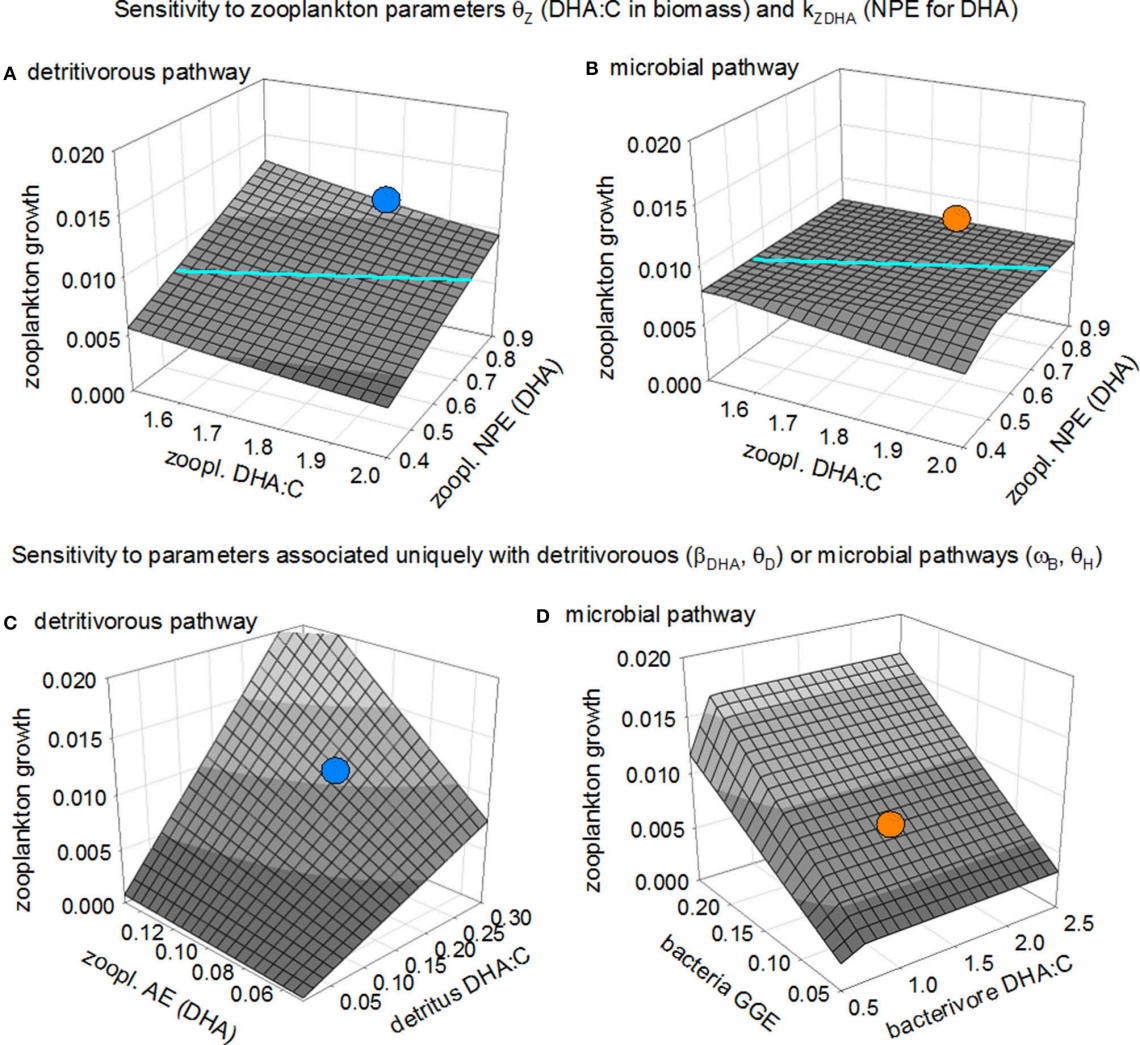
**Sensitivity of predicted zooplankton growth to parameters θ_***Z***_ (zooplankton DHA:C ratio; mmol mol^**−1**^) and ***k***_***ZDHA***_ (zooplankton NPE of DHA) for the detritivorous and microbial pathways (panels A and B; the colored lines demarcate where the two planes intersect) and sensitivity to key parameters associated with the two pathways: (C)** detritivorous pathway, parameters θ_*D*_ (detritus DHA:C ratio) and β_*ZDHA*_ (zooplankton absorption efficiency for DHA in detritus) and **(D)** microbial pathway, parameters θ_*H*_ (bacterivore DHA:C ratio) and ω_*B*_ (B GGE). The two blue points indicate predicted growth following the detritivorous pathway as shown in Figure 2, and the two orange points the corresponding predicted growth following the microbial pathway.

We used parameter sensitivity analysis to investigate the circumstances under which predicted zooplankton growth is greatest following the microbial pathway. Figures 3A,B illustrate how chosen parameter values for zooplankton net production efficiency for DHA (*k*_*ZDHA*_) and the DHA:C in zooplankton biomass (θ_*Z*_) influence growth following the two pathways. Zooplankton are DHA-limited in the detritivorous pathway throughout the parameter domain (Figure 3A). Recent work has shown that a range of aquatic invertebrates, including marine zooplankton, catabolize essential PUFAs at high rates (Mezek et al., 2010; Mayor et al., 2011, 2015; Maity et al., 2012) in which case our default zooplankton NPE for DHA of 0.9 (Anderson and Pond, 2000; Mayor et al., 2009) may be too high. Reducing the value of this parameter results in a proportional lowering of predicted zooplankton growth, to the extent that the detritivorous pathway becomes an inferior source of nutrition relative to the microbial pathway (in areas of the plane shown in Figure 3A that are lower than those of the corresponding parameter space shown in Figure 3B). Increasing the DHA:C ratio in the biomass of zooplankton, thereby increasing the demand for DHA, likewise causes a decrease in predicted growth following the detritivorous pathway. Growth following the microbial pathway is, in contrast, relatively insensitive to changing either *k*_*ZDHA*_ or θ_*Z*_ throughout most of the parameter space because limitation is by C (Figure 3B).

Figures 3C,D show the sensitivity of zooplankton growth to the absorption efficiency for DHA (β_*ZDHA*_) and the detritus DHA:C ratio (θ_*D*_) for the detritivorous pathway, and bacterial gross growth efficiency (ω_*B*_) and DHA:C ratio in protistan bacterivores (θ_*H*_) for the microbial pathway. Predicted growth following the detritivorous pathway is limited by DHA and so declines as this micronutrient becomes less available, either due to decreased absorption efficiency and/or reduced availability in detritus (Figure 3C). Our default value for the DHA:C of detritus (θ_*D*_ = 0.21 mmol DHA mol C^−1^) is likely too high because the samples upon which it is based were from a relatively shallow depth and did not exclude microbes from the detrital matter (see “Data sources” section), leading to overestimated growth following the detritivorous pathway. We assumed that C and DHA within detritus are absorbed by zooplankton with the same efficiency (β_*ZC*_ = β_*ZDHA*_ = 0.1), i.e., these animals are unable to selectively extract DHA from the detritus matrix. If they were able to do so, which is achieved in the model by increasing parameter β_*ZDHA*_ while keeping β_*ZC*_ at 0.1, the detritivorous pathway then becomes more profitable as a source of nutrition (Figure 3C). Growth of zooplankton following the microbial pathway shows no sensitivity to the DHA:C ratio in protistan bacterivores, except when this ratio is very low (<0.7; Figure 3D) because, although the bacterivores are a plentiful supply of DHA, limitation is by C. Growth does, however, increase with increasing bacterial growth efficiency because this results in more C being incorporated into the microbial food web.

In summary, the sensitivity analysis presented in Figure 3 confirms the findings of Figure 2, showing the basic trade-off facing detritivorous zooplankton: a choice between consuming high quantity, low quality detritus via the detritivorous pathway which leads to limitation by DHA, or a low quantity, high quality protistan diet via the microbial pathway, with limitation by C. The analysis of Figure 2 showed that, with the default parameter set, the growth of zooplankton was greatest following the detritivorous pathway. The trade-off choice of opting for DHA-rich microbes (the microbial pathway) was less favorable in this instance because the losses of C due to trophic transfer in the microbial food web overrode the gains in greater DHA availability. The sensitivity analysis showed that this situation can easily be reversed by alteration of various parameter values, leading to the microbial pathway being the superior source of nutrition for zooplankton: predicted growth via the detritivorous pathway decreased when the net production efficiency for DHA (*k*_*ZDHA*_) or the DHA:C in detritus (θ_*D*_) are lowered, or when the DHA:C of zooplankton biomass (θ_*Z*_) was increased. Increasing bacterial gross growth efficiency (ω_*B*_), which promotes protistan growth, also reduced the relative effectiveness of the detrital pathway. On the other hand, the detritivorous pathway became a better source of nutrition if zooplankton were assumed to selectively absorb DHA from detritus (increase in β_*ZDHA*_ relative to β_*ZC*_). We conclude that, given uncertainty associated with these various parameters, it is currently impossible to say with any certainty that either pathway will necessarily provide the best source of nutrition for detritivorous zooplankton in the MPZ of the ocean. The analysis has nevertheless highlighted that the microbial pathway, i.e., trophic upgrading, has the potential to be the best source of nutrition in many instances, based on results for the combinations of parameters investigated in the sensitivity analysis.

**Figure 4:**
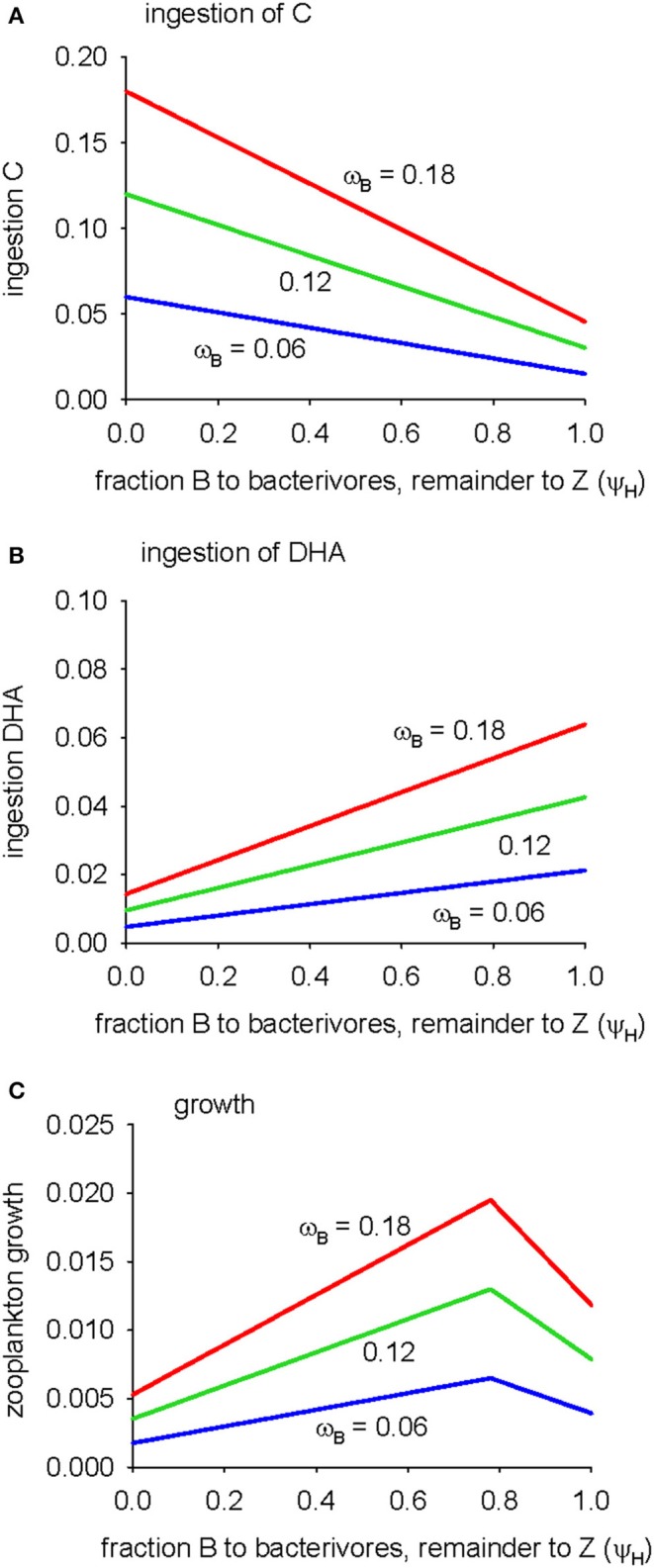
**Sensitivity of zooplankton growth via the microbial pathway to parameter ψ_**H**_ (the fate of bacteria: fraction ψ_**H**_ to flagellates and fraction 1-ψ_**H**_ to zooplankton; standard value (Table 2) is ψ_**H**_ = 1), for B GGE (parameter ω_***B***_) = 0.06, 0.12, 0.18: (A)** ingestion of C, **(B)** ingestion of DHA, **(C)** growth.

The analysis of the microbial pathway has thus far assumed that 100% of bacterial losses are due to grazing by protistan bacterivores (ψ_*H*_ = 1) and that bacteria do not therefore contribute to the diet of detritivorous zooplankton. Decreasing this parameter short-circuits the microbial food chain as fraction (1-ψ_*H*_) of bacteria are then consumed directly by zooplankton. Taken to the extreme (ψ_*H*_ = 0), all bacteria go to zooplankton. The effects of increasing the proportion of bacteria directly ingested by zooplankton (0 ≤ ψ_*H*_ ≤ 1) on predicted ingestion of C and DHA following the microbial pathway, and the resulting zooplankton growth, are shown in Figure 4. Bacteria constitute the base of the microbial food web and so direct access to this food source (low values of ψ_*H*_), rather than the bacterivores one trophic level above, increases the C available to zooplankton (Figure 4A). On the other hand, bacterial biomass has a low DHA:C ratio and so the quantity of ingested DHA decreases as the proportion of bacteria ingested by zooplankton increases (low ψ_*H*_; Figure 4B). A point is reached, ψ_*H*_ = 0.78, where the supply of C and DHA is optimal and growth is maximized (Figure 4C). Growth is limited by C for ψ_*H*_ > 0.78 and by DHA for ψ_*H*_ < 0.78, respectively. Increasing bacterial gross growth efficiency (parameter ω_*B*_) supplies extra DHA and C via the microbial pathway but does not influence the ratio of bacterial growth to bacterivore growth in the microbial food web and therefore has no effect on the optimum dietary intake of bacterial biomass (ψ_*H*_). Overall, the analysis of Figure 4 shows that C-limitation of zooplankton growth via the microbial pathway can be alleviated if these animals are able to access bacteria directly as a food source.

**Figure 5:**
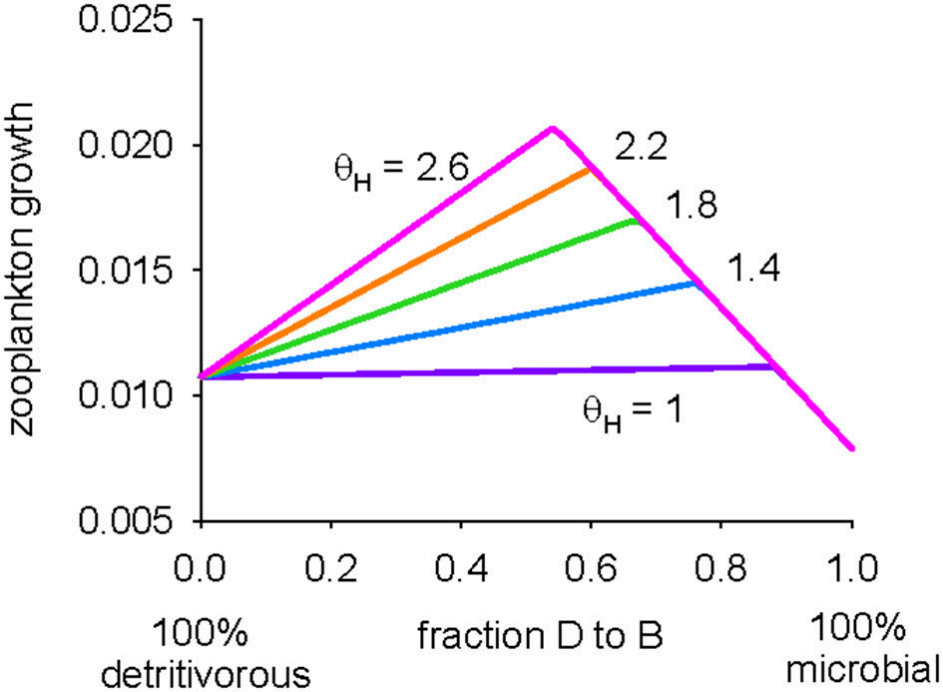
**Predicted zooplankton growth for 0 (pure detritivorous) ≤ ψ_**B**_ ≤ 1 (pure microbial pathway) and θ_***H***_ (DHA:C ratio in protistan bacterivores) between 1.0 and 2.6 mmol mol^**−1**^**.

We conclude our analysis of the model by moving away from examining the detritivorous and microbial pathways in isolation from each other, and look at zooplankton growth when the two pathways are utilized simultaneously. In other words, rather than examining the two end members, the detrital pathway (ψ_*B*_ = 0) and microbial pathway (ψ_*B*_ = 1), growth is now shown for the full range, 0 ≤ ψ_*B*_ ≤ 1 (Figure 5). The growth of zooplankton is maximized when the diet consists of a mix of detritus and protistan bacterivores, irrespective of the bacterivore DHA:C ratio (θ_*H*_). The growth of these copepods is limited by C to the right of the optimum because of C losses in the microbial food web, whereas limitation is by DHA to the left because of the low DHA content in detritus. Increasing the bacterivore DHA:C ratio offsets DHA limitation and thus increases the requirement for C in detritus in order to achieve optimal nutrition (and so the optimum ψ_*B*_ shifts to the left). Assuming that the DHA:C ratio in protistan bacterivores (θ_*H*_) is 1.4 (Table 2), growth is maximized when ψ_*B*_ is 0.76, indicating that the optimal diet is primarily microbial.

## Discussion

A new model is presented and used herein to investigate the nutrition of metazoan detritivores, specifically the trade-off between consuming a diet of high-quantity, low-quality detritus versus a low-quantity, high quality diet that is rich in nutritious microbial biomass. The study focuses on the MPZ of the open ocean and involves a stoichiometric analysis of the growth of metazoan zooplankton with model currencies of C, because of its role in structural biomass and energy provisioning, and DHA, which is central to physiological adaptations to the cold temperatures and high pressures typical of the MPZ (Hazel and Williams, 1990). The model extends our previous C-only flow analysis (Mayor et al., 2014) that examined the potential gains that mesopelagic zooplankton stand to make from promoting and subsequently harvesting microbial growth via the fragmentation of large detrital particles, so-called “microbial gardening” (Fenchel, 1970). The model here was first used to compare the growth of zooplankton when consuming a diet consisting solely of non-living detritus (the “detritivorous pathway”) versus growth when consuming a purely microbial diet (the “microbial pathway”). The microbial pathway represents “trophic upgrading” (Klein Breteler et al., 1999) of the non-living detrital substrate, i.e., consumption of the community of micronutrient-rich protistan bacterivores that colonize detritus, but which are present in low biomass because of losses in trophic transfer within the microbial food web. The conditions which maximize the growth of zooplankton were subsequently examined, where both detritus and microbes are utilized simultaneously in a mixed diet.

Our initial comparison of the two pathways, detritivorous and microbial, showed that predicted zooplankton growth could, at least in theory, be higher on the former (Figure 2). The nutritional benefits of consuming microbes were offset by the increased potential for zooplankton to be limited by food quantity (C). We assumed that zooplankton only had access to the protistan bacterivores in our baseline calculations, with no consumption of bacteria. The movements of motile protists, such as the myriad flagellates that colonize sinking marine detritus (Patterson et al., 1993; Turner, 2002), indicate that they should be readily detected by mechanoreceptors that are typical to copepods (Kiørboe, 2011). If zooplankton consume a diet consisting of protistan bacterivores, much of the detrital C is lost to bacterial and protistan respiration within the particle-attached microbial loop (Azam et al., 1983). This facet of the model underscores the need to understand the dynamics of microbial food webs and their interaction with higher trophic levels.

The limitation of zooplankton growth by food quantity (C) following the microbial pathway can be alleviated if direct ingestion of bacteria is possible. This short-circuits the microbial loop, removing losses of C through protistan respiration, but also lowering the DHA content of the ingested ration because the DHA:C content of bacterial biomass is considerably lower than that of their protistan predators (see Data Sources section). The potential for limitation by DHA therefore becomes more acute under this scenario, although the optimum ratio between the size of copepods of their prey (18:1; Hansen et al., 1994) suggests that direct and deliberate ingestion of bacteria by zooplankton (0.1-1 mm) is unlikely. Another possible short circuit of the microbial pathway occurs if the protists in our model are allowed to directly consume detritus (e.g., Poulsen et al., 2011). This shortening of the food chain between detritus and zooplankton via the microbial pathway is more favorable for zooplankton growth, relative to the bacteria short circuit, because the protists are rich in DHA. It follows that understanding the efficiency and structure of the microbial loop, and the trophic level at which detritivorous consumers interact with this food web, are both crucial for the development of quantitative models to explore the biogeochemistry of detrital ecosystems.

Further exploration of the model involving parameter sensitivity analysis highlighted a range of conditions where the microbial pathway is more favorable than the detritivorous pathway as a source of zooplankton nutrition. Increasing bacterial growth efficiency beyond its standard value of 0.12 is perhaps the most obvious way to achieve this, thereby directly increasing the flow of C into the microbial food web. Reported BGEs are highly variable and often very low (Steinberg et al., 2008). The stoichiometric prediction of zooplankton growth also depends heavily on the DHA:C ratios in seston used in the calculation. These are not well known for the MPZ. Our default value for the ratio in detritus may be somewhat high because the underlying data were derived from measurements in the upper MPZ using methods that did not distinguish between detritus and the associated detrital community (see Data Sources section). Decreasing this ratio, or increasing the DHA:C ratio in zooplankton biomass, both lead to the microbial pathway becoming more favorable than the detritivorous pathway. A further assumption in the model parameterization is that zooplankton can utilize DHA with high efficiency (*k*_*ZDHA*_ = 0.9; Table 2), i.e., this essential micronutrient is solely required for physiological adaptations and is not used for energy generation (Anderson and Pond, 2000; Mayor et al., 2009). Recent observations suggest, however, that at least some marine copepods have high metabolic demands for DHA and other PUFAs (Mayor et al., 2011, 2015) and thus utilize these compounds with relatively low efficiency. Lowering the assumed efficiency with which DHA is utilized increases the demand for this essential fatty acid and so is another way of increasing the potential for the microbial pathway to be a superior source of nutrition to the detritivorous pathway. We are unaware of any data that specifically relates to the demands for DHA or other micronutrients in mesopelagic copepods and call for observations and experiments that may generate such information.

The idea that microbes support the growth of higher trophic levels is not new. An early study found that a detritus-consuming amphipod, *Parhyalella whelpleyi*, obtains its nutrition from the associated microbial communities, the non-living plant residue passing undigested through the gut (Fenchel, 1970). Stream invertebrates have also been observed to preferentially feed on leaves that have been colonized and “conditioned” by microorganisms (Kaushik and Hynes, 1971; Bärlocher and Kendrick, 1975). The nutritional environment facing detritivores has been likened to humans eating peanut butter and crackers (Cummins, 1974), microbial biomass being akin to the nutritious peanut butter spread on the indigestible crackers. Following on from this early work, a number of studies have since shown microbial biomass to be a potentially important source of nutrition for invertebrates in a range of systems including deposit-feeding mayflies (Edwards and Meyer, 1990; Hall and Meyer, 1998), leaf shredders (Connolly and Pearson, 2013), benthic polychaetes (Gontikaki et al., 2011), earthworms (Larsen et al., 2016) and other soil animals including collembolans, mites, woodlice and centipedes (Pollierer et al., 2012; Lemanski and Scheu, 2014). Recent observations have even revealed potentially important trophic linkages between detritus-associated microbes and vertebrates such as fish (e.g., Choy et al., 2015). Given the global importance of heterotrophic protists in the MPZ of the ocean (Pernice et al., 2015) and their role in biosynthesizing essential micronutrients such as DHA (Zhukova and Kharlamenko, 1999), we suggest that these organisms are highly likely to feature in the diets of metazoans that reside in this habitat.

Analysis of zooplankton ingesting a mixture of pure detritus and protistan biomass (Figure 5) showed that it may be that the optimal diet involves utilization of both the detritivorous and microbial pathways in combination, with C supplied by the former balanced by DHA from the microbes. The predicted optimal diet using the standard parameter set (Table 2) contained a strong microbial component (the detritivorous and microbial pathways contributed 24 and 76% respectively to nutrition; ψ_*B*_ = 0.76). The analysis thus demonstrates the potential for protistan biomass to be the primary, if not the sole, part of the diet of metazoan zooplankton (Mayor et al., 2014), although this result is of course subject to the uncertainties in predicted growth highlighted by the parameter sensitivity analyses shown in Figures 3, 4. Both our study and that of Mayor et al. (2014) achieve this result, at least in part, because they are underpinned by the assumption that energy and nutrients within detritus are absorbed with much lower efficiencies than those in microbial biomass, i.e., flagellates and other soft bodied protists are more easily digested than detrital particles consisting of refractory compounds such as cellulose and chitin. We are unaware of any empirical data to directly verify this assumption, but it is supported by the conspicuous absence of flagellate remains in the guts and feces of zooplankton (reviewed by Turner, 2002), despite their long-since acknowledged significance as prey items (Stoecker and Capuzzo, 1990). We further reason that it is likely harder for zooplankton to digest and absorb detrital material, particularly as particles sink deeper into the oceans interior, because it is continuously reworked and repackaged by heterotrophic organisms that strip out anything of energetic or nutritional value (Podgórska and Mundryk, 2003; Wilson et al., 2008). The effects of this stripping are manifest as declining particulate concentrations of nitrogen and micronutrients such as fatty acids and amino acids with increasing water depth (Wakeham et al., 1997; Fileman et al., 1998; Schneider et al., 2003). An improved knowledge of the efficiencies with which mesopelagic zooplankton process different food items is required in order to further our quantitative understanding of the flows of energy and organic matter in detrital food webs. This is a particularly challenging task, potentially requiring the need for *in situ* experiments that determine absorption efficiencies and food preferences for a range of detritivorous invertebrates.

Evolving the means for internal digestion of recalcitrant organic compounds represents a stark alternative to encouraging, or even allowing, microbial growth on external particles of detritus. Recent work on terrestrial detritivores has highlighted a plethora of intricate relationships between invertebrates and their microbiome that facilitate the internal digestion of lignocellulose and other refractory molecules (König and Varma, 2006). In termites, for example, digestion of refractory material is achieved through symbiotic relationships with both bacteria and flagellates (Bignell et al., 2011; Brune, 2014). Relationships of this kind typically require the presence of one or more enlarged gut compartments to house specific microbial communities that carry out fermentation under anoxic conditions (Plante et al., 1990), such as the voluminous hindgut paunch observed in termites (Brune and Dietrich, 2015). The apparent absence of specialized gut structures in copepods commonly found in the mesopelagic, e.g., *Oithona* spp. and *Oncaea* spp., and their small size (≤1 mm) relative to typical detritivorous invertebrates on land (>10 mm), suggest that internal digestive symbioses are not particularly prevalent in midwater crustaceans. Indeed, the conspicuous difference in size between detritivorous invertebrates in terrestrial and mesopelagic ecosystems may arise because the evolutionary pressures to remain small (Kiørboe, 2011) outweigh the need for internal microbially-mediated fermentation in particle-collecting marine zooplankton. More effort is required to identify the internal microbiome of mesopelagic copepods and understand its physiological roles.

Marine detritivorous zooplankton, including *Oithona*, contain significant levels of DHA (Kattner et al., 2003; Pond and Ward, 2011) and numerous studies have highlighted the physiological roles of unsaturated fatty acids in adaptations to temperature and pressure (Cossins and Macdonald, 1989; Hazel and Williams, 1990; Pond et al., 2014). It was assumed that detritivorous invertebrates in our model have physiological requirements for DHA that cannot be met by endogenous biosynthesis, either by the copepods or their internal microbiome, i.e., DHA is an essential micronutrient. The potential for endogenous DHA biosynthesis in detritivorous copepods, by contrast, remains equivocal. Work on benthic copepods suggests that these animals may be capable of elongating shorter-chain PUFA [e.g., 18:3(n-3)] into DHA (Norsker and Støttrup, 1994; Nanton and Castell, 1998; De Troch et al., 2012), but this is not the case for epipelagic zooplankton (Bell et al., 2007). Terrestrial invertebrates are reported to obtain essential micronutrients such as amino acids and fatty acids via their biosynthesis by gut microbes (e.g., Sampedro et al., 2006; Brune, 2014) but the extent to which this occurs in marine invertebrates remains unclear (Plante et al., 1990; Harris, 1993). The guts of marine copepods are known to harbor bacteria (Sochard et al., 1979), some of which show potential for PUFA biosynthesis (Jøstensen and Landfald, 1997), but their actual role(s) within these organisms remains poorly understood. Indeed, we can find no clear evidence that marine copepods are capable of endogenous DHA biosynthesis in the absence of pre-cursor PUFAs, as we propose would be necessary for mesopelagic copepods consuming refractory detritus alone. New information on the source(s) of DHA and other micronutrients in mesopelagic detritivores will provide useful insight into the ecology and biogeochemistry of their habitat. Advances in this area may arise from examining the isotopic signatures of specific micronutrient compounds in detritivores and comparing these to the values found in autotrophic producers and mesopelagic detritus. Improved understanding of the biosynthetic capabilities of animals from the mesopelagic and the significance of internal microorganisms, potentially arising through the application of genomic, transcriptomic and metabolomic techniques, will further help resolve this knowledge gap.

In conclusion, our results indicate that ingesting nutrient-rich microbial biomass potentially represents a beneficial strategy relative to consuming refractory detritus, despite the considerable losses of C due to the inefficiency of the microbial loop. Overall, our work has highlighted how little we know about the physiology of the organisms within detritivorous food webs and hence how and why they interact with organic matter and the wider ecosystem. “Despite their global distribution and essential roles in nutrient cycling, microbial decomposers are among the least known organisms in terms of elemental concentrations and stoichiometric relationships” (Danger et al., 2016). We suggest that better understanding the ecology and physiology of organisms in the mesopelagic is urgently required if we are to develop mechanistic biogeochemical models of this important ecosystem.

## Author contributions

TA led the work, developing the model and generating the results, with a major contribution from DM in terms of advising on parameterizations, data, and in the analysis and writing of the manuscript. DP provided additional advice, especially regarding the fatty acid work described in the manuscript.

### Conflict of interest statement

The authors declare that the research was conducted in the absence of any commercial or financial relationships that could be construed as a potential conflict of interest.
